# Comparison of AI-based radiographic interpretation versus endodontic specialists for identifying periapical lesions: An *in vitro* study

**DOI:** 10.6026/973206300214831

**Published:** 2025-12-15

**Authors:** Snehal Gosavi, Jasmine Marwaha, Naif Omar Binmuhana, Santhosh Kumar Caliaperoumal, Bassam Alkhalifah, Mohammed Mustafa

**Affiliations:** 1 Department of Conservative Dentistry and Endodontics, Al badar dental college and hospital, Gulbarga, Karnataka 585102, India; 2 Department of Conservative dentistry and Endodontics, National Dental College and Hospital, Mohali-140507, India; 3 Department of Preventive Dental Science, Division of Pediatric Dentistry, Ibn Sina National College for Medical Studies, Jeddah, Saudi Arabia; 4 Department of dentistry, Vinayaka Mission's Medical College and hospital, Vinayaka Mission's Research Foundation (Deemed to be University), Karaikal - 609 609, Puducherry, India; 5 Department of Radiology, College of Medicine, Qassim University, Buraydah, Saudi Arabia; 6 Department of Conservative Dental Sciences, College Of Dentistry, Prince Sattam Bin Abdulaziz University, Al-Kharj 11942, Saudi Arabia

**Keywords:** Artificial intelligence, deep learning, convolutional neural network, periapical lesions, endodontics, diagnostic accuracy, digital radiography

## Abstract

The detection of periapical lesion is a diagnostic problem that sometimes entails the use of experts who can correctly interpret the radiographs to be accurate. Therefore, it is of interest to compare an AI system that uses CNN with endodontic experts in the classification of periapical lesions on 500 digital periapical radiographs. The AI was sensitive, specific and accurate with 89.2, 91.5, and 90.2 respectively, which is close to the performance of specialists and is able to process images more than 20 times faster. There were no differences in diagnostic measures of AI and experts. Thus, we show that AI-aided radiographic analysis may be used as a safe, time-saving supplement to the endodontic practice to detect periapical lesions.

## Background:

Proper diagnosis of periapical pathology is one of the most important elements of an endodontic practice that has direct effect on the choice of treatment planning and patient outcomes [1]. Periapical radiography is still the most used mode of imaging in the detection and follow-up assessment of periapical lesions despite the limitations such as two-dimensional visualization of three-dimensional object and the variability in interpretation [2]. Accuracy in the diagnosis of periapical lesions among different practitioners is highly variable, as it also depends on the level of experience, the condition of viewing, and the level of individual perceptions [3]. The recent achievements in artificial intelligence (AI), specifically, deep learning algorithms based on convolutional neural networks (CNNs), have shown impressive results in the medical image analysis field in several fields [4]. In dentistry, AI-based systems have been created to complete a number of diagnostic works such as detecting caries, reviewing bone loss periodontitis, and locating anatomical landmarks [5]. AI-based endodontic radiographic interpretation is an area of the future, which can possibly provide objective, standardized, and quick diagnostic assistance [6]. A number of preliminary studies have examined AI use in identifying periapical lesion with promising outcomes [7]. The machine learning algorithms have demonstrated the ability to differentiate between normal and diseased periapical tissues, detecting opaque radiographic changes that cannot be visualized [8]. Nevertheless, the majority of current research has used datasets that are not very large, did not compare them with interpretation at the specialist level, or used simplified tasks of binary classification [9]. The gold standard diagnostic test of periapical pathology is usually a histopathological test, but cone-beam computed tomography (CBCT) has a higher imaging quality than traditional radiography [10]. However, there are practical and economical aspects that require the continuity of the use of periapical radiography as a routine clinical screening method. Determining whether AI systems can perform as well in terms of diagnostic outcome as trained specialists is a very important step towards clinical deployment [11]. With a long period of training and clinical practice, endodontic specialists build sensitive diagnostic skills in interpreting the periapical lesions [12]. The analysis of AI activity against the level of expertise of specialists is a demanding account of determining clinical utility. In addition, the knowledge of inter-specialist diagnostic variability places AI performance in the range of human diagnostic accuracy [13]. Although the use of AI in dentistry is increasingly popular, there are still large knowledge gaps in terms of oral health care needs comparative diagnostic performance of advanced CNN-based applications and endodontics professionals in detecting periapical lesions [14]. There is also a paucity of data on the performance of the AI system under different lesion sizes, regions of the anatomy, and different radiographic quality conditions [15]. Careful consideration of these factors is the responsibility to be considered prior to clinical integration being recommended. Therefore, it is of interest to conduct a rigorous comparison of the diagnostic accuracy, sensitivity, specificity, and efficiency of a CNN-based AI system and board-certified endodontic specialists in detecting the presence of periapical lesions on the digital periapical radiographs.

## Materials and Methods:

## Study design and ethical approval:

This retrospective diagnostic accuracy study was conducted at the Department of Endodontics, University Dental Institute, between January 2024 and September 2024. Digital periapical radiographs were retrospectively collected from the institutional database spanning 2018-2022. A total of 500 periapical radiographs of permanent teeth were selected based on predefined criteria.

## Inclusion criteria:

Digital periapical radiographs of permanent teeth (anterior, premolar, and molar regions); adequate technical quality (proper angulation, sufficient density, no motion artifacts); confirmed diagnostic status through CBCT imaging and clinical examination within 30 days of periapical radiograph; patients aged 18-75 years.

## Exclusion criteria:

Radiographs with severe technical defects (underexposure, overexposure, geometric distortion); images showing endodontic treatment in progress; presence of extensive restorations obscuring periapical region; radiographs of developing teeth with incomplete root formation; previous periapical surgery; severe periodontal bone loss complicating periapical assessment. The dataset comprised 287 radiographs with confirmed periapical lesions and 213 radiographs without periapical pathology (healthy controls). Periapical lesions were defined as radiolucent areas associated with tooth apices measuring ≥2mm in diameter, confirmed by CBCT examination showing bone destruction and clinical symptoms or signs. All radiographs were acquired using standardized digital imaging systems (Schick 33, Sirona Dental Systems, or VistaScan Mini, Dürr Dental) with consistent exposure parameters.

## Reference standard establishment:

The reference standard (ground truth) was established through comprehensive evaluation combining: (1) CBCT imaging performed within 30 days of periapical radiograph showing three-dimensional confirmation of periapical bone changes; (2) clinical findings including percussion sensitivity, palpation tenderness, sinus tract presence, and pulp vitality testing; (3) consensus diagnosis by two senior endodontists not participating in the evaluation phase, based on integrated CBCT and clinical data. Cases with disagreement underwent third-party adjudication.

## Endodontic specialist evaluation:

Six board-certified endodontic specialists (mean experience: 12.8 ± 4.3 years post-specialty certification) independently evaluated all 500 radiographs in randomized order. Specialists were blinded to clinical information, CBCT findings, and AI results. Each specialist reviewed radiographs in three separate sessions (maximum 200 images per session) separated by minimum 72 hours to minimize fatigue effects. Radiographs were displayed on calibrated 27-inch diagnostic monitors (resolution: 2560x1440 pixels) in dimly lit rooms. Specialists used proprietary viewing software allowing zoom, brightness, and contrast adjustments. For each radiograph, specialists indicated: (1) presence or absence of periapical lesion; (2) confidence level (5-point Likert scale: 1=very uncertain, 5=very certain); (3) approximate lesion size if present (small: 2-5mm, medium: 5-10mm, large: >10mm); (4) tooth number and anatomical location. Evaluation time per image was recorded.

## AI system development and architecture:

A custom CNN-based AI system was developed using Python 3.8 with TensorFlow 2.9 framework. The network architecture comprised a modified ResNet-50 backbone with transfer learning, incorporating the following components:

## Training dataset:

The AI system was trained on 15,000 annotated periapical radiographs collected from multiple institutions (separate from the 500-image test set). Training data included 9,200 images with periapical lesions and 5,800 without lesions, with expert-verified annotations.

## Data augmentation:

Training utilized augmentation techniques including random rotation (±15°), horizontal flipping, brightness adjustment (±20%), contrast variation (±15%), and Gaussian noise addition to improve generalization.

## Network architecture:

The model consisted of convolutional layers for feature extraction, global average pooling, dropout layers (rate: 0.5) for regularization, and fully connected layers culminating in sigmoid activation for binary classification. The network contained approximately 23.5 million trainable parameters.

## Training protocol:

The model was trained for 150 epochs using Adam optimizer (learning rate: 0.0001), binary cross-entropy loss function, and batch size of 32. Training was performed on NVIDIA Tesla V100 GPU. Validation set (3,000 images) monitored overfitting, with early stopping implemented.

## Output:

For each test image, the AI system provided: (1) binary classification (lesion present/absent); (2) probability score (0-1 scale); (3) heatmap visualization highlighting regions contributing to classification decision; (4) processing time.

## Evaluation protocol:

Both specialists and AI system evaluated the identical 500-image test set. The test set was completely independent from AI training and validation data. Image presentation order was randomized differently for each specialist to minimize bias. AI evaluation was conducted after all specialist assessments were completed to ensure complete blinding.

## Outcome measures:

## Primary outcomes:

[1] Sensitivity (true positive rate)

[2] Specificity (true negative rate)

[3] Overall diagnostic accuracy

[4] Positive predictive value (PPV)

[5] Negative predictive value (NPV)

[6] Area under the receiver operating characteristic curve (AUC-ROC)

## Secondary outcomes:

[1] Diagnostic performance stratified by lesion size

[2] Performance across different tooth types

[3] Inter-rater reliability among specialists (Fleiss' kappa)

[4] Agreement between AI and specialists (Cohen's kappa)

[5] Processing time per image

[6] Confidence levels and their correlation with accuracy

## Statistical analysis:

Statistical analyses were performed using R version 4.2.1 (R Foundation for Statistical Computing) and MedCalc version 20.1. Descriptive statistics included mean ± standard deviation for continuous variables and frequencies (percentages) for categorical variables. Sensitivity, specificity, accuracy, PPV, and NPV were calculated with 95% confidence intervals using the Clopper-Pearson method. ROC curves were generated, and AUC values were calculated for AI and each specialist. DeLong's test compared AUC values between AI and mean specialist performance. Independent t-tests compared continuous variables between groups. Chi-square tests evaluated categorical outcome differences. Inter-rater reliability was assessed using Fleiss' kappa for multiple specialists and Cohen's kappa for pairwise comparisons. Kappa interpretation followed Landis and Koch criteria (<0.20=poor, 0.21-0.40=fair, 0.41-0.60=moderate, 0.61-0.80=substantial, 0.81-1.00=almost perfect). Subgroup analyses examined diagnostic performance across lesion sizes (small, medium, large) and tooth types (anterior, premolar, molar). Mixed-effects logistic regression models evaluated factors influencing diagnostic accuracy. Statistical significance was set at p<0.05 (two-tailed). Sample size calculation indicated that 500 radiographs provided 90% power to detect 5% difference in sensitivity/specificity with α=0.05.

## Results:

The final dataset comprised 500 digital periapical radiographs representing 178 anterior teeth (35.6%), 147 premolars (29.4%), and 175 molars (35.0%). Among the 287 radiographs with periapical lesions, 94 (32.8%) were classified as small (2-5mm), 136 (47.4%) as medium (5-10mm), and 57 (19.8%) as large (>10mm) based on CBCT measurements. The 213 control radiographs showed no evidence of periapical pathology on both CBCT and clinical evaluation. Table 1 (see PDF) presents the comprehensive diagnostic performance metrics for the AI system and individual endodontic specialists. No statistically significant differences were observed between AI system and mean specialist performance for any primary outcome measure. The AI system demonstrated sensitivity of 89.2% compared to mean specialist sensitivity of 91.4% (p=0.183). Specificity was 91.5% for AI versus 88.7% for specialists (p=0.142). Overall accuracy was nearly identical: 90.2% for AI and 90.3% for specialists (p=0.934). Table 2 (see PDF) presents diagnostic performance stratified by lesion size and anatomical location. Diagnostic performance improved significantly with increasing lesion size for both AI and specialists. Small lesions (2-5mm) were detected with 76.6% sensitivity by AI and 78.2% by specialists, while large lesions (>10mm) achieved >98% sensitivity for both. No significant performance differences were observed between AI and specialists across any lesion size category (p>0.05 for all comparisons). Regarding tooth type, anterior teeth showed slightly higher detection rates (AI: 91.4%, Specialists: 93.8%) compared to premolars (AI: 88.1%, Specialists: 90.3%) and molars (AI: 87.9%, Specialists: 89.7%), though differences were not statistically significant. The AI system maintained comparable performance to specialists across all anatomical locations. Table 3 (see PDF) presents inter-rater reliability metrics and processing time comparisons. Inter-specialist agreement demonstrated substantial concordance (Fleiss' κ=0.742), indicating good consistency among experts. Agreement between AI and individual specialists ranged from κ=0.729 to κ=0.781 (mean: 0.754), also representing substantial agreement and comparable to inter-specialist reliability. Processing time differed dramatically, with AI requiring 0.8 ± 0.2 seconds per image compared to 18.3 ± 4.7 seconds for specialists (p<0.001), representing approximately 23-fold faster analysis. When AI confidence scores exceeded 0.9 (68.4% of cases), diagnostic accuracy reached 95.6%, comparable to specialists' accuracy at maximum confidence level (96.8%). Detailed error analysis revealed that AI false negatives (31 cases) primarily involved small lesions in posterior regions with complex anatomical overlapping structures. Specialists' false negatives (mean: 24.7 cases) showed similar patterns. AI false positives (18 cases) predominantly occurred in areas with anatomical radiolucencies (mental foramen, maxillary sinus, incisive canal) and widened periodontal ligament spaces. Specialists' mean false positives (24.0 cases) showed comparable distribution.

## Discussion:

This comprehensive *in vitro* experiment proved that a CNN-based AI system performed at least as well in diagnostic tests compared to board certified endodontic specialists to detect periapical lesions on computerized periapical radiographs, and has significantly lower processing time. The sensitivity (89.2%), specificity (91.5%), and accuracy (90.2) of the AI system were similar to the endodontic mean specialist performance of all measures, which contribute to the possible clinical application of AI as a diagnostic aid in endodontics. The observed diagnostic accuracy of the proposed study is consistent with the previous studies that examine the application of AI in dental radiographic interpretation [1]. Other researchers have described inconsistent AI detection of periapical lesions, with sensitivity of 78 to 95-percent depending on the characteristics of the dataset, algorithm architecture, and definition of lesions [2]. Our findings are in the higher percentile of reported performance which is probably due to the advanced CNN architecture, large training set (15,000 images), and stringent data augmentation protocols used [3]. The similarity in the performance of AI and specialists is specifically remarkable because endodontic specialists are the clinical standard of gerund and interpretation of radiographs. Pattern recognition, anatomy, and synthesis of radiographic results with the clinical setting are the main components of the specialist training [4]. The fact that AI systems can reach the same level of diagnostic accuracy points to the successful computational modeling of expert-level patterns of visual analysis but the underlying cognitive mechanisms are different in nature [5]. The lesion size stratified performance showed the anticipated trends, with both the AI and the specialists performing better in terms of sensitivity at larger lesions. Even small periapical lesions (2-5mm) posed diagnostic difficulties to both AI (76.6% sensitivity) and specialists (78.2% sensitivity) due to the inherent weakness of the two-dimensional radiography to detect subtle changes in the bone. This observation highlights the fact that AI systems, similar to human interpreters are limited by the content of information in the source images [7]. The introduction of AI into clinical practice must recognize these constraints and take suitable diagnostic care over the equivocal results. The high level of agreement between specialists (Fleiss κ=0.742) is a good evidence that specialists do agree, nevertheless, the level of diagnostic variability is still high even among the specialists. Such variability is the subjectivity of radiograph interpretation and depends on perceptual thresholds, risk tolerance, and personal experience [8]. AI-specialist agreement (mean 0.754) was similar to inter-specialist agreement and this implies that AI brings new diagnostic variability comparable to introducing another expert to make assessments as opposed to a systematic deviation difference based on the specialist decision [9]. The time difference in the processing (0.8 seconds on AI and 18.3 seconds on specialists) is dramatic, which is an important practical benefit on its own. This improvement in efficiency may be of significant benefit in workflow in high-volume clinical or screening environments [10]. Nevertheless, AI systems are not to substitute thorough clinical examination, but they can assist in obtaining valuable answers in a short period of time. This accuracy was high when AI confidence had high values of 0.9 (95.6), which has led to the possible existence of confidence-based triage systems, where cases with high AI confidence are expedited and uncertain cases are further reviewed by specialists [11]. The error pattern analysis showed that both AI and experts had difficulties with the following diagnosis issues: small posterior lesions and atypical anatomy and normal radiolucent pathology. Such common patterns of errors imply that some radiographic appearances have potentially ambiguous aspects, which frustrate both computer and human determination [12]. The next step in AI development may involve the use of anatomical landmark identification to differentiate between pathologic radiolucencies and normal structures, which may lower the false positive rate [13]. These findings have a number of significant clinical consequences. To begin with, AI systems that are capable of performing well enough in the role of specialists may have useful purposes in the context of limited specialist availability, offering a standardized method of assisting general dentists with diagnostic services [14]. Second, AI may serve as a second reader model, which alerts specific lesions and which require specialist confirmation and lowers the number of missed pathology. Third, AI systems do not have fatigue effects and provide high-level performance, which may be of use in large-scale screening programs or quality assurance applications [15].

Significant drawbacks should be mentioned. First, it was an *in vitro* study that examined the images of radiographs in a statical form, without clinical context which strongly commonly informs a diagnostic decision. Pulp testing, periapical diagnosis, and patient history play a significant part in the practice of periapical diagnosis. Second, the reference standard applied to CBCT and clinical results instead of histopathological confirmation which is the actual gold standard. Nonetheless, histopathological confirmation cannot be applied to large scale studies and CBCT ensures a great quality of imaging in bone pathology. Third, quality of all radiographs was sufficient; in real practice with worse quality images may be observed. Fourth, the AI system was applied to one institutional dataset; external validation in various populations and imaging systems would be valuable to enhance claims of generalizability. Fifth, the research was conducted on binary classification (lesion present/absent) and not more subtle diagnostic groupings (granuloma versus cyst, acute versus chronic). The possible future research directions will include a number of questions. There is a need to conduct clinical studies on the AI effect on the quality of diagnosis and treatment decision-making in clinical practice. Clinical utility would increase with investigation of AI performance to monitor longitudinal lesion and assess healing. Comprehensive diagnostic assistance can be offered by developing multi-task AI systems that would be able to evaluate various pathologies and the quality of treatment simultaneously, as well as identify anatomical landmarks. AI-CBCT analysis will be able to address the drawbacks of two-dimensional imaging. Lastly, the cost-effectiveness studies on AI-assisted versus traditional diagnostic workflows would guide the implementation decisions.

## Conclusion:

The CNN-based AI system demonstrated diagnostic accuracy, sensitivity, and specificity equivalent to board-certified endodontic specialists for periapical lesion detection while operating far faster. Its consistent performance across lesion types highlights its reliability as a supportive diagnostic tool. AI can enhance efficiency and accessibility in endodontic diagnosis when used to complement, not replace, expert clinical judgment.
